# Decreased Interleukin-1 Family Cytokine Production in Patients with Nontuberculous Mycobacterial Lung Disease

**DOI:** 10.1128/spectrum.03110-22

**Published:** 2022-10-18

**Authors:** Bock-Gie Jung, Kristin Dean, Carly Wadle, Buka Samten, Deepak Tripathi, Richard J. Wallace, Barbara A. Brown-Elliott, Torry Tucker, Steven Idell, Julie V. Philley, Ramakrishna Vankayalapati

**Affiliations:** a Department of Pulmonary Immunology, The University of Texas Health Science Center at Tyler, Tyler, Texas, USA; b Department of Microbiology, The University of Texas Health Science Center at Tyler, Tyler, Texas, USA; c Department of Cellular and Molecular Biology, The University of Texas Health Science Center at Tyler, Tyler, Texas, USA; d The Texas Lung Injury Institute, Tyler, Texas, USA; e Department of Medicine, The University of Texas Health Science Center at Tyler, Tyler, Texas, USA; Johns Hopkins University School of Medicine

**Keywords:** nontuberculous mycobacteria, pulmonary disease, TWIK2, interleukin-1

## Abstract

Nontuberculous mycobacteria (NTM) cause pulmonary disease in individuals without obvious immunodeficiency. This study was initiated to gain insight into the immunological factors that predispose persons to NTM pulmonary disease (NTMPD). Blood was obtained from 15 pairs of NTMPD patients and their healthy household contacts. Peripheral blood mononuclear cells (PBMCs) were stimulated with the Mycobacterium avium complex (MAC). A total of 34 cytokines and chemokines were evaluated in plasma and PBMC culture supernatants using multiplex immunoassays, and gene expression in the PBMCs was determined using real-time PCR. PBMCs from NTMPD patients produced significantly less interleukin-1β (IL-1β), IL-18, IL-1α, and IL-10 than PBMCs from their healthy household contacts in response to MAC. Although plasma RANTES levels were high in NTMPD patients, they had no effect on IL-1β production by macrophages infected with MAC. Toll-like receptor 2 (TLR2) and TWIK2 (a two-pore domain K+ channel) were impaired in response to MAC in PBMCs of NTMPD patients. A TLR2 inhibitor decreased all four cytokines, whereas a two-pore domain K+ channel inhibitor decreased the production of IL-1β, IL-18, and IL-1α, but not IL-10, by MAC-stimulated PBMCs and monocytes. The ratio of monocytes was reduced in whole blood of NTMPD patients compared with that of healthy household contacts. A reduced monocyte ratio might contribute to the attenuated production of IL-1 family cytokines by PBMCs of NTMPD patients in response to MAC stimulations. Collectively, our findings suggest that the attenuated IL-1 response may increase susceptibility to NTM pulmonary infection through multiple factors, including impaired expression of the TLR2 and TWIK2 and reduced monocyte ratio.

**IMPORTANCE** Upon MAC stimulation, the production of IL-1 family cytokines and IL-10 by PBMCs of NTMPD patients was attenuated compared with that of healthy household contacts. Upon MAC stimulation, the expression of TLR2 and TWIK2 (one of the two-pore domain K+ channels) was attenuated in PBMCs of NTMPD patients compared with that of healthy household contacts. The production of IL-1 family cytokines by MAC-stimulated PBMCs and MAC-infected monocytes of healthy donors was reduced by a TLR2 inhibitor and two-pore domain K+ channel inhibitor. The ratio of monocytes was reduced in whole blood of NTMPD patients compared with that of healthy household contacts. Collectively, our data suggest that defects in the expression of TLR2 and TWIK2 in human PBMCs or monocytes and reduced monocyte ratio are involved in the reduced production of IL-1 family cytokines, and it may increase susceptibility to NTM pulmonary infection.

## INTRODUCTION

Nontuberculous mycobacteria (NTM) refer to all members of the genus Mycobacterium except for the Mycobacterium tuberculosis complex, which causes tuberculosis (TB), and Mycobacterium leprae and Mycobacterium lepromatosis, which causes leprosy (1–3). NTM can cause disseminated disease in patients with immunodeficiencies, whether primary or acquired, but these organisms cause mainly isolated pulmonary disease in individuals without obvious immunodeficiency (3, 4). The Mycobacterium avium complex (MAC), which consists mainly of M. avium and Mycobacterium intracellulare, is the most frequently isolated group in patients with NTM pulmonary diseases (NTMPDs) (3).

Recently, the incidence and prevalence of NTMPD have increased worldwide. In the United States from 2008 to 2015, the annual incidence of NTMPD increased from 3.13 to 4.73 per 100,000 person/years, and the annual prevalence increased from 6.78 to 11.70 per 100,000 persons (5). These trends are consistent with other studies from Europe (6), Japan (7), and South Korea (8). Although immunological risk factors that cause NTM disseminated infection have been well studied in immunocompromised hosts, there is no clear information on how NTMPD develops in immunocompetent hosts.

NTM disseminated infection is associated with a reduction in CD4^+^ T cells in patients with AIDS (9) and a deficiency in genes affecting the interleukin-12 (IL-12) and interferon-γ (IFN-γ) pathways in patients with Mendelian susceptibility to mycobacterial disease (MSMD) (4). However, there is no evidence of a reduction in CD4^+^ T cells in patients with NTMPD (10). IL-12 and IFN-γ responses in patients with NTMPD have been inconsistent, with reports of increased (11), unchanged (10), or decreased (12–14) levels. Similar inconsistencies have been reported regarding the role of tumor necrosis factor-α (TNF-α) in patients with NTMPD. Some studies have reported attenuated TNF-α responses (12, 14, 15), but others found an intact TNF-α response (10, 13) in patients with NTMPD.

To gain insight into the immunologic factors that contribute to the development of NTMPD, we evaluated the immune responses of NTMPD patients and their healthy household contacts. We found defective IL-1β, IL-18, IL-1α, and IL-10 production by patient peripheral blood mononuclear cells (PBMCs) in response to MAC antigens. We also determined the mechanisms responsible for the defective production of these cytokines.

## RESULTS

### Cytokine and chemokine production by PBMCs of NTMPD patients and healthy household contacts in response to MAC antigens.

**FIG 1:**
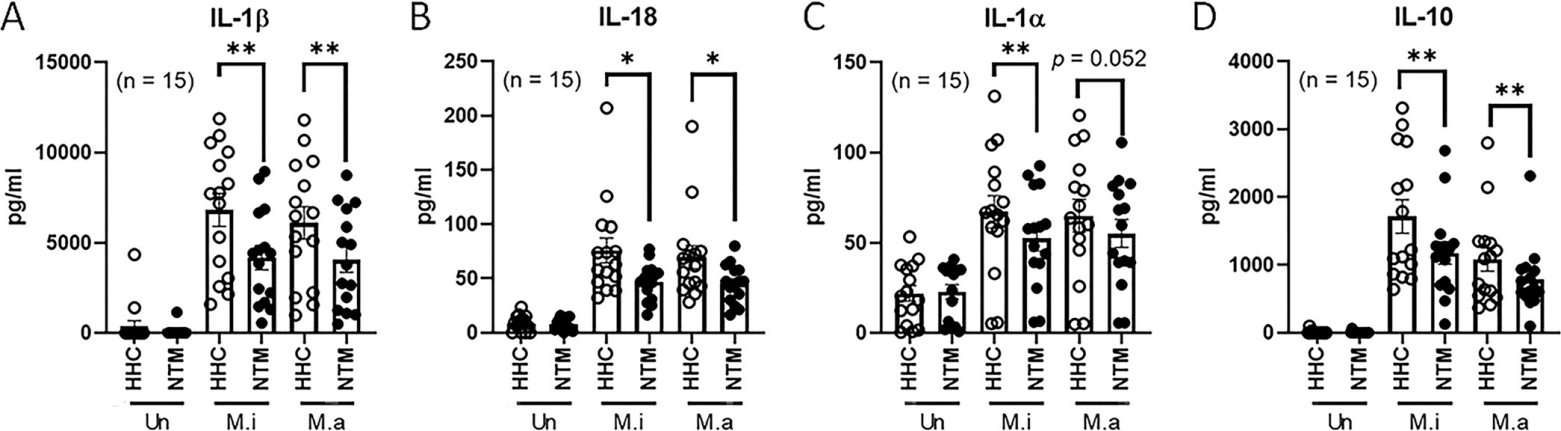
Production of cytokines and chemokines by M. avium complex (MAC)-stimulated peripheral blood mononuclear cells (PBMCs). PBMCs were isolated from 15 pairs of NTMPD patients (NTM) and healthy household contacts (HHC) and cultured in the presence or absence of 10 μg/mL heat-killed MAC (M. intracellulare [M.i] or M. avium [M.a]) for 72 h. The concentrations of 34 cytokines and chemokines were measured by multiplex immunoassay (Fig. S1). Data for cytokines interleukin-1β (IL-1β) (A), IL-18 (B), IL-1α (C), and IL-10 (D) are shown. Data are expressed as the means, and error bars represent SEM. Each individual circle (open circle, healthy household contacts; closed circle, NTMPD patients) indicates an individual person. *, *P < *0.05; and **, *P < *0.01.

We cultured PBMCs from 15 NTMPD patients and 15 healthy household contacts with heat-killed MAC antigens. After 72 h, culture supernatants were collected, and 34 cytokine and chemokine levels were measured by multiplex immunoassay (see Fig. S1 in the supplemental material). PBMCs from NTMPD patients produced significantly less IL-1β, IL-18, IL-1α, and IL-10 than PBMCs from their healthy household contacts in response to heat-killed MAC antigens (Fig. 1). In response to heat-killed M. intracellulare antigens, PBMCs of NTMPD patients produced 4,187.0 ± 681.1 pg/mL IL-1β, 46.9 ± 4.3 pg/mL IL-18, 52.5 ± 7.0 pg/mL IL-1α, and 1,178.0 ± 167.9 pg/mL IL-10, whereas PBMCs of their healthy household contacts produced 6,819.0 ± 895.7 pg/mL IL-1β (*P < *0.01) (Fig. 1A), 76.0 ± 11.5 pg/mL IL-18 (*P < *0.05) (Fig. 1B), 67.2 ± 8.9 pg/mL IL-1α (*P < *0.01) (Fig. 1C), and 1,715.0 ± 242.0 pg/mL IL-10 (*P < *0.01) (Fig. 1D). Similarly, in response to heat-killed M. avium antigens, PBMCs of NTMPD patients produced 4,073.0 ± 698.6 pg/mL IL-1β, 45.4 ± 4.6 pg/mL IL-18, 55.2 ± 7.6 pg/mL IL-1α, and 789.0 ± 126.2 pg/mL IL-10, whereas PBMCs of their healthy household contacts produced 6,105.0 ± 883.9 pg/mL IL-1β (*P < *0.01) (Fig. 1A), 69.7 ± 10.7 pg/mL IL-18 (*P < *0.05) (Fig. 1B), 65.0 ± 9.1 pg/mL IL-1α (*P* = 0.052) (Fig. 1C), and 1,082.0 ± 174.4 pg/mL IL-10 (*P < *0.01) (Fig. 1D). Our findings demonstrate that PBMCs of NTMPD patients produce less IL-1β, IL-18, IL-1α, and IL-10 in response to MAC antigens than PBMCs of their healthy household contacts.

### Cytokine and chemokine levels in the plasma of patients and healthy household contacts.

**FIG 2:**
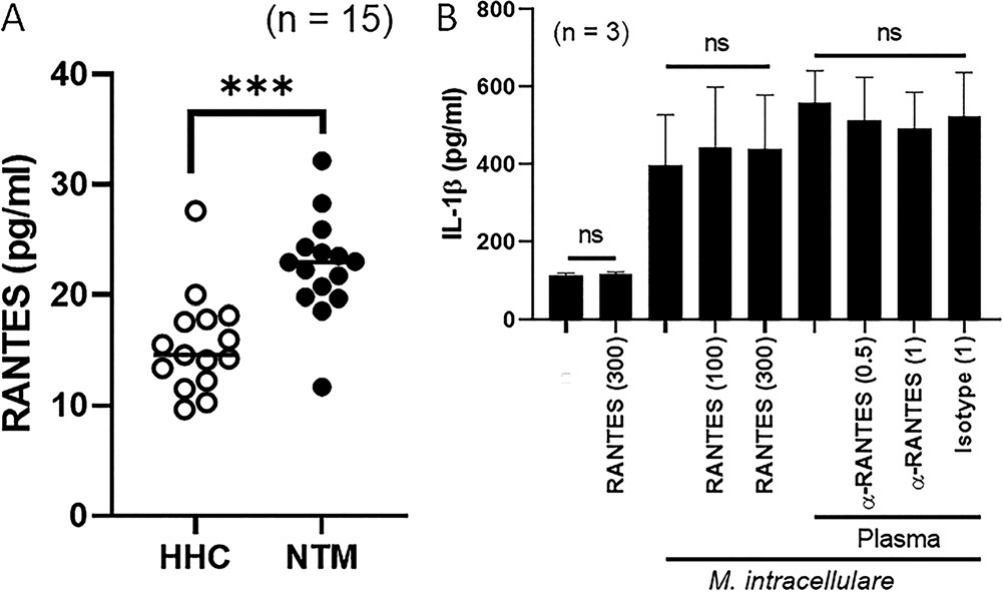
Plasma RANTES levels and their effect on IL-1β production by macrophages. Plasma samples were collected from 15 pairs of NTMPD patients (NTM) and their healthy household contacts (HHC). Concentrations of 34 cytokines and chemokines were measured by multiplex immunoassay (Fig. S2) and data for RANTES (A) are shown. Monocyte-derived macrophages (MDM) from 3 healthy volunteers were infected with M. intracellulare at a multiplicity of infection (MOI) of 20 in the presence or absence of RANTES (100 or 300 ng/mL) or plasma from NTMPD patients (10 μL of plasma was pretreated with or without anti-RANTES antibody [0.5 or 1 μg/mL] or isotype antibody) for 24 h. The concentration of IL-1β was measured by ELISA (B). Data are expressed as each individual circle (open circle, healthy household contacts; closed circle, NTMPD patients) and line (median value) (A). Data are expressed as the means, and error bars represent SEM (B). ***, *P < *0.001; ns, not significant.

We also measured cytokine and chemokine levels by multiplex immunoassay in the plasma of patients and healthy household contacts (see Fig. S2 in the supplemental material). Among 34 cytokines and chemokines, RANTES levels were significantly higher in NTMPD patient plasma (22.6 ± 1.2 pg/mL) than those in healthy household contact plasma (15.5 ± 1.2 pg/mL) (*P < *0.001) (Fig. 2A).

### Effect of RANTES on IL-1β production by macrophages.

RANTES is required for IFN-γ production by T cells (16) and promotes nitric oxide production by macrophages (17). The essential role of IL-1β in the protection against mycobacterial diseases has been well demonstrated (18–20). Since RANTES was the only chemokine significantly elevated in the plasma of patients, we determined whether RANTES can affect IL-1β production by macrophages. Monocyte-derived macrophages (MDM) were infected with M. intracellulare and incubated in the presence of various concentrations of recombinant RANTES or plasma from NTMPD patients (in some cases, plasma was pretreated with an anti-RANTES antibody or isotype antibody) at 37°C in 5% CO_2_. Cell culture supernatants were collected after 24 h of incubation, and IL-1β concentrations were measured by enzyme-linked immunosorbent assay (ELISA) (Fig. 2B). M. intracellulare-induced IL-1β (397.1 ± 130.8 pg/mL) was not significantly changed by recombinant RANTES (443.0 ± 155.8 pg/mL at 100 ng/mL RANTES, 440.5 ± 138.4 pg/mL at 300 ng/mL RANTES) or plasma from NTMPD patients (559.0 ± 82.5 pg/mL after addition of plasma, 512.2 ± 112.1 pg/mL after addition of plasma pretreated with 0.5 ng/mL anti-RANTES antibody, 492.3 ± 94.0 pg/mL after addition of plasma pretreated with 1 ng/mL anti-RANTES antibody, 524.3 ± 112.9 pg/mL after addition of plasma pretreated with 1 μg/mL of isotype control antibody). These data suggest that RANTES has no effect on IL-1β production by macrophages infected with M. intracellulare.

### NTMPD patients have an impaired expression of TLR2 and TWIK2.

**FIG 3:**
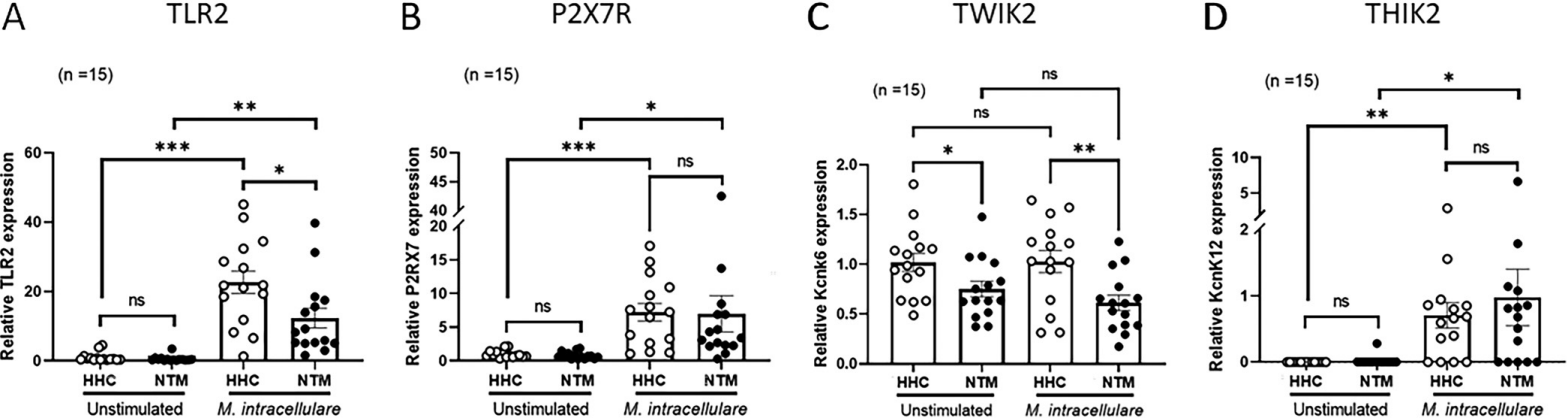
TLR2 (A), P2X7R (B), TWIK2 (C), and THIK2 (D) expression in M. intracellulare-stimulated PBMCs. PBMCs were isolated from 15 pairs of NTMPD patients (NTM) and healthy household contacts (HHC), and the cells were cultured in the presence or absence of 10 μg/mL heat-killed M. intracellulare for 72 h. Gene expression was measured by real-time PCR using 18S as an internal control with specific primer and probe sets (Table S1). TREK1 signals were not detected in any samples until 40 cycles of real-time PCR. Data are expressed as the means, and error bars represent SEM. Each individual circle (open circle, healthy household contacts; closed circle, NTMPD patients) indicates an individual person. *, *P < *0.05; **, *P < *0.01; and ***, *P < *0.001. ns, not significant.

Toll-like receptor 2 (TLR2), P2X7R receptor (P2X7R), and TWIK2 are involved in mycobacterium-induced proinflammatory signaling, especially IL-1β production (21–23). To determine whether any of these receptors and channels are involved in reduced IL-1β, IL-18, IL-1α, and IL-10 production, we cultured PBMCs of NTMPD patients and their healthy household contacts with or without heat-killed M. intracellulare. We determined the relative mRNA expression levels of TLR2, P2X7R, and two-pore domain potassium (K+) efflux channels, including TWIK2, THIK2, and TREK1 (Fig. 3). Upon stimulation with heat-killed M. intracellulare antigens, the relative mRNA expression of TLR2 and TWIK2 in PBMCs was significantly reduced in NTMPD patients compared with that of their healthy household contacts (Fig. 3A and C). In contrast, there was no difference in the expression of P2X7R and THIK2 in these PBMCs. (Fig. 3B and D). Of note, the relative mRNA expression level of TWIK2 in PBMCs was significantly reduced in NTMPD patients compared with that of their healthy household contacts even without stimulation (Fig. 3C). TREK1 expression was not detected in any samples until 40 cycles of real-time PCR amplification (data not shown). These data suggest that the expression of TLR2 and TWIK2 is impaired in NTMPD patients.

### TLR2 inhibition reduces IL-1β, IL-18, IL-1α, and IL-10 production by PBMCs and monocytes in response to MAC.

**FIG 4:**
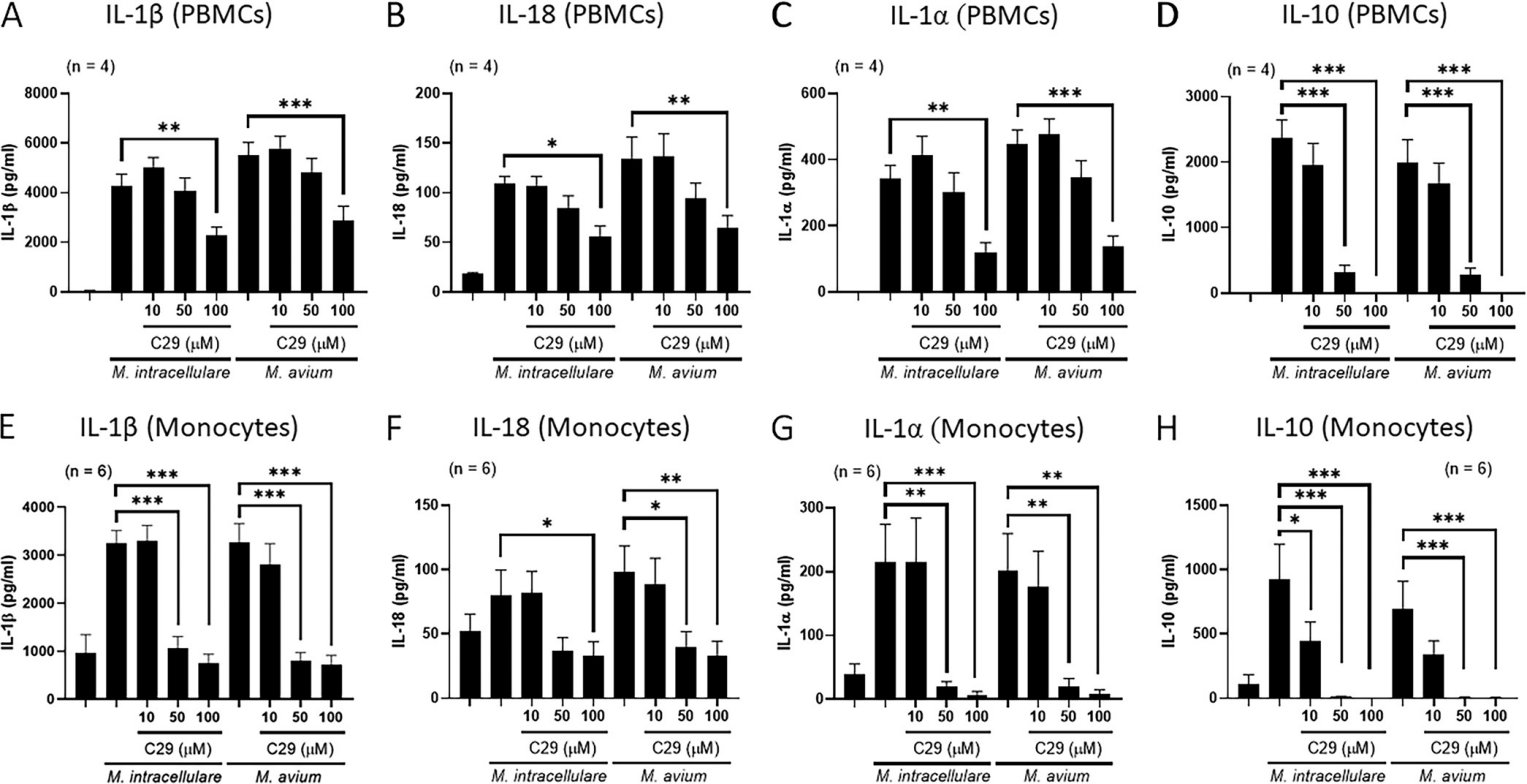
Effect of a TLR2 inhibitor on the production of IL-1β, IL-18, IL-1α, and IL-10 by PBMCs and CD14^+^ monocytes stimulated with M. avium complex (MAC). PBMCs from 4 healthy volunteers were stimulated with 10 μg/mL heat-killed MAC antigens in the presence or absence of various concentrations (10, 50, and 100 μM) of C29 (a TLR2-selective inhibitor) for 24 h (A to D). Monocytes of 6 healthy volunteers were infected with live MAC at an MOI of 10 in the presence or absence of various concentrations (10, 50, and 100 μM) of C29 (a TLR2-selective inhibitor) for 18 h (E to H). The concentrations of cytokines were measured by ELISA. Data are expressed as the means, and error bars represent SEM. *, *P < *0.05; **, *P < *0.01; and ***, *P < *0.001. ns, not significant.

We next determined whether the reduced expression of TLR2 is related to the reduced production of IL-1β, IL-18, IL-1α, and IL-10 by PBMCs stimulated with heat-killed MAC antigens. PBMCs from 4 healthy donors were cultured with or without heat-killed MAC antigens in the presence or absence of a TLR2-selective inhibitor (C29). The TLR2-selective inhibitor significantly reduced IL-1β, IL-18, IL-1α, and IL-10 production by heat-killed MAC antigen-stimulated PBMCs in a dose-dependent manner (Fig. 4A to D). Monocytes and macrophages are known as the major sources of IL-1β, IL-18, and IL-1α. We next determined whether TLR2 is involved in the production of IL-1β, IL-18, IL-1α, and IL-10 by monocytes infected with live MAC. Monocytes from 6 healthy donors were infected with or without MAC in the presence or absence of a TLR2-selective inhibitor (C29). The TLR2-selective inhibitor significantly reduced the production of IL-1β, IL-18, IL-1α, and IL-10 by MAC-infected CD14^+^ monocytes in a dose-dependent manner (Fig. 4E to H). The TLR2-selective inhibitor (C29) had no effect on cell viability (see Fig. S3A and B in the supplemental material). These data suggest that TLR2 is involved in IL-1β, IL-18, IL-1α, and IL-10 production by PBMCs and monocytes in response to MAC.

### Two-pore domain K+ channel inhibition reduces IL-1β, IL-18, and IL-1α but not IL-10 production by PBMCs and monocytes in response to MAC.

**FIG 5:**
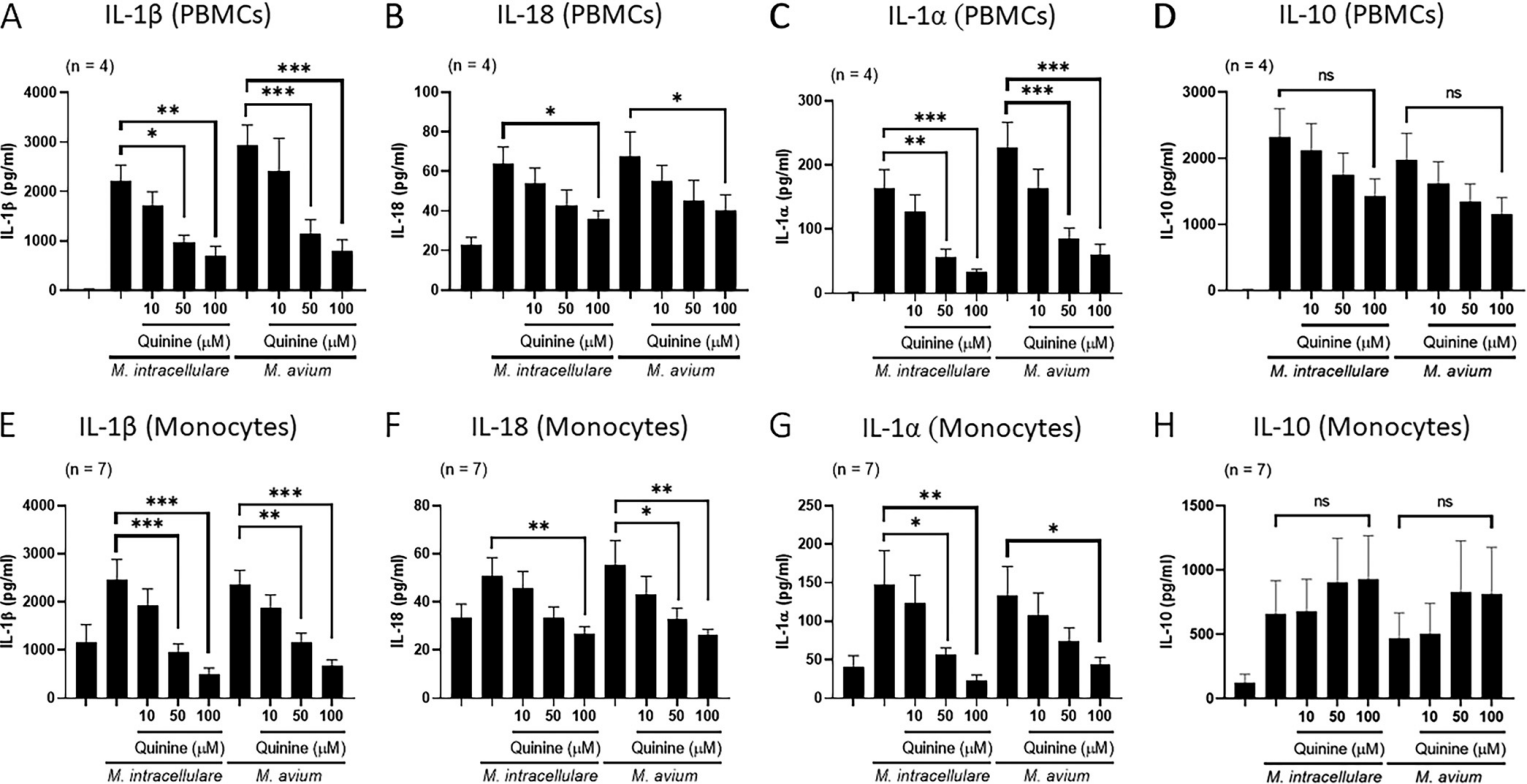
Effect of a two-pore domain K+ channel inhibitor on the production of IL-1β, IL-18, IL-1α, and IL-10 by PBMCs and CD14^+^ monocytes stimulated with M. avium complex (MAC). PBMCs from 4 healthy volunteers were stimulated with 10 μg/mL heat-killed MAC antigens in the presence or absence of various concentrations (10, 50, and 100 μM) of quinine (a two-pore domain K+ channel inhibitor) for 24 h (A to D). Monocytes of 7 healthy volunteers were infected with live MAC at an MOI of 10 in the presence or absence of various concentrations (10, 50, and 100 μM) of quinine (a two-pore domain K+ channel inhibitor) for 18 h (E to H). The concentrations of cytokines were measured by ELISA. Data are expressed as the means, and error bars represent SEM. *, *P < *0.05; **, *P < *0.01; and ***, *P < *0.001. ns, not significant.

We next determined whether the reduced expression of the two-pore domain K+ channel is related to the reduced production of IL-1β, IL-18, IL-1α, and IL-10 by PBMCs stimulated with heat-killed MAC antigens. PBMCs from 4 healthy donors were cultured with or without heat-killed MAC antigens in the presence or absence of a two-pore domain K+ channel inhibitor (quinine). Quinine significantly reduced IL-1β, IL-18, and IL-1α, but not IL-10, production by heat-killed MAC antigen-stimulated PBMCs in a dose-dependent manner (Fig. 5A to D). We next determined whether a two-pore domain K+ channel is involved in the production of IL-1β, IL-18, IL-1α, and IL-10 by monocytes infected with live MAC. Monocytes from 7 healthy donors were infected with or without MAC in the presence or absence of quinine. Quinine significantly reduced IL-1β, IL-18, and IL-1α, but not IL-10, production by MAC-infected CD14^+^ monocytes in a dose-dependent manner (Fig. 5E to H). The two-pore domain K+ channel inhibitor (Quinine) had no effect on cell viability (see Fig. S3C and D in the supplemental material). These data suggest that the two-pore domain K+ channel is involved in IL-1β, IL-18, and IL-1α, but not IL-10, production by PBMCs and monocytes in response to MAC.

### Immune cell subpopulations in whole blood of NTMPD patients and their healthy household contacts.

**FIG 6:**
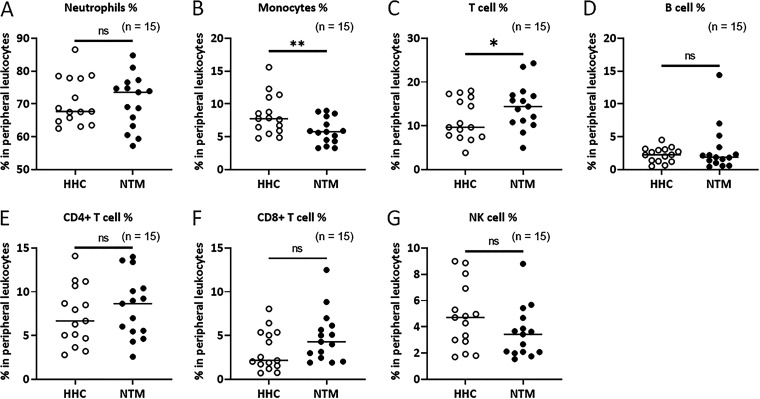
Immune cell subpopulation in peripheral blood. To compare the ratio of the peripheral blood leukocyte subpopulation between NTMPD patients and their own healthy household contacts, heparinized whole blood was stained with various antibodies (Table S2). FlowJo software version 10 (BD) was used for flow cytometry data analysis. The gating strategy for leukocyte subpopulations in human whole peripheral blood is shown in Fig. S4. Data are expressed as the means, and error bars represent SEM. Each individual circle (open circle, healthy household contacts, closed circle, NTMPD patients) indicates an individual person. *, *P < *0.05; and **, *P < *0.01. ns, not significant.

We also identified immune cell subpopulations in the whole blood of patients and healthy household contacts using flow cytometry with various antibodies (Fig. 6). The ratio of monocytes was reduced in whole blood of NTMPD patients (5.90% ± 0.51%) compared with that of their healthy household contacts (8.34% ± 0.79%) (*P < *0.01) (Fig. 6B), whereas the ratio of CD3^+^ T lymphocytes was increased in whole blood NTMPD patients (14.46% ± 1.35%) compared with that of their healthy household contacts (11.45% ± 1.21%) (*P < *0.05) (Fig. 6C). There were no significant differences between NTMPD patients and healthy household contacts in the ratio of other immune cell subpopulations, including neutrophils (patients versus healthy 70.73% ± 2.11% versus 70.83% ± 1.89%), B cells (3.06% ± 0.93% versus 2.27% ± 0.29%), CD4^+^ T cells (8.26% ± 0.93% versus 7.33% ± 0.88%), CD8^+^ T cells (4.86% ± 0.76% versus 3.28% ± 0.59%), and natural killer (NK) cells (3.56% ± 0.51% versus 4.77% ± 0.64%).

## DISCUSSION

Limited information is available on the immune responses of patients with NTMPD (3). In the current study, we compared the immune responses of NTMPD patients and their healthy household contacts in response to NTM antigens. Our findings demonstrate that in response to MAC antigens, PBMCs from NTMPD patients produced less IL-1β, IL-18, IL-1α, and IL-10 and that TLR2 and TWIK2 expression was reduced compared with those of their healthy household contacts. We also found that the inhibition of TLR2 decreased the production of IL-1β, IL-18, IL-1α, and IL-10 by PBMCs and monocytes from healthy donors in response to MAC, whereas inhibition of the two-pore domain K+ channel decreased the production of IL-1β, IL-18, and IL-1α, but not IL-10, by PBMCs and monocytes from healthy donors in response to MAC.

The essential role of IL-12, IFN-γ, and TNF-α in the host defense against TB (24, 25) and NTM disseminated disease (3, 4) is well documented. However, there are conflicting reports about their role in NTMPD (10–15). In the current study, we found that PBMCs from NTMPD patients and their healthy household contacts produced similar amounts of IL-12, IFN-γ, and TNF-α in response to MAC antigens (see Fig. S1 in the supplemental material). IL-1 family cytokines (IL-1β, IL-18, and IL-1α) produced by antigen-presenting cells are known to play an important role against various pathogens, including M. tuberculosis (18–20). Similarly, PBMCs and macrophages from NTMPD patients produce less IL-1β and IL-18 than those from healthy donors in response to MAC antigen (26) or lipopolysaccharide (LPS) (10). However, the factors responsible for the reduced IL-1β levels in PBMCs from NTMPD patients are unknown. Our current results confirm the above findings and further identify a possible mechanism for the reduced production of IL-1β, IL-18, and IL-1α by macrophages of NTMPD patients.

Plasma RANTES levels were significantly higher in NTMPD patients than those in the plasma of their healthy household contacts, similar to previous findings in Mycobacterium abscessus- and M. tuberculosis-infected patients (27, 28). RANTES is a potent chemoattractant factor for monocytes and T cells and is associated with the function of cytotoxic T cells, which are important in the control of various intracellular pathogens (29). RANTES polymorphisms are associated with an increased risk of TB (30–32). In the current study, we found that recombinant RANTES protein or plasma RANTES did not significantly alter IL-1β production by macrophages (Fig. 2B), suggesting that increased levels of RANTES are not responsible for the reduced IL-1β production by macrophages.

IL-10 is a potent anti-inflammatory cytokine and is well known as a deleterious cytokine during infection with various pathogens mainly via deactivation of macrophages, resulting in diminished Th1 cytokine production (33). There are conflicting reports on IL-10 production by PBMCs of NTMPD patients in response to MAC antigens. PBMCs from NTMPD patients produce more IL-10 than PBMCs from healthy donors in response to MAC antigens (heat-killed M. intracellulare) (12), tuberculin and sensitin (11); other studies found no significant difference between NTMPD patients and healthy subjects (10, 14). Another study found that whole blood from NTMPD patients produced less IL-10 than healthy donors in response to LPS, heat-killed Staphylococcus epidermidis, or live M. intracellulare (13). In addition to IL-1β, IL-18, and IL-1α, PBMCs from NTMPD patients produced less IL-10 in response to MAC antigens (Fig. 1D).

A two-step mechanism is required for the production and release of IL-1 family cytokines, such as IL-1β by macrophages (34). First, transcription and translation of the inactive form of IL-1β are initiated from the recognition of pathogen-associated molecular patterns (PAMPs) by pattern recognition receptors (PRRs), such as TLRs. Second, assembly of the inflammasome is induced by further signals such as K+ efflux. This assembled inflammasome complex induces the maturation and release of IL-1β (35). TLRs, especially TLR2, play an important role in the innate host response to mycobacteria, leading to the production of cytokines (21). P2X7R plays a central role in the maturation and secretion of IL-1β by activating ATP-dependent K+ efflux (35). A recent study suggested that TWIK2 (a member of the two-pore domain K+ efflux channel family) activates P2X7R-independent K+ efflux and mediates the production of IL-1β in macrophages (36). We found a reduced expression of TLR2 and TWIK2, but not P2X7R, THIK2, or TREK1, in PBMCs of NTMPD patients compared with that of their healthy household contacts. Inhibition of TLR2 expression on PBMCs and monocytes of healthy donors at the time of MAC antigen stimulation or MAC infection reduced IL-1β, IL-18, IL-1α, and IL-10 production, whereas inhibition of a two-pore domain K+ channel by PBMCs and monocytes of healthy donors at the time of MAC antigen stimulation or MAC infection reduced IL-1β, IL-18, and IL-1α, but not IL-10, production. Our findings suggest that impaired expression of TLR2 can lead to the defective production of IL-1β, IL-18, IL-1α, and IL-10 in NTMPD patients. However, impaired expression of a two-pore domain K+ channel is responsible for the defective production of IL-1β, IL-18, and IL-1α, but not IL-10, in NTMPD patients.

A previous study showed that TLR2 gene polymorphisms are associated with the development of NTMPD (26, 37). The TWIK2 K+ efflux channel mediates NLRP3 inflammasome-induced inflammation in macrophages (36), and an attenuated NLRP3 inflammasome is correlated with decreased IL-1β responses and host susceptibility in NTMPD patients (26). However, activation of the NLRP3 inflammasome by M. tuberculosis is uncoupled from susceptibility to active TB (38). These findings may suggest that TWIK2-mediated NLRP3 inflammasome activation is involved in host defense against NTMPD but not TB. Therefore, future studies to understand the precise mechanisms associated with TWIK2-mediated NLRP3 inflammasome activation in NTMPD are needed to develop immunomodulatory strategies to treat or prevent NTMPD.

Since different ratios of immune cell subpopulations may contribute to the different levels of cytokine production by PBMCs in response to MAC stimulations, we also identified immune cell subpopulations in the whole blood of NTMPD patients and healthy household contacts. The ratio of monocytes was reduced in whole blood of NTMPD patients compared with that of their healthy household contacts. Since monocytes and macrophages are known as the major sources of IL-1 family cytokines (39), a reduced monocyte ratio might be one of the reasons why the productions of IL-1β, IL-18, and IL-1α are significantly less by PBMCs of NTMPD patients compared with those of healthy household contacts in response to MAC stimulations. Increased ratios of CD3^+^ T lymphocytes may be because of the reduced ratio of monocytes in whole blood of NTMPD patients compared with those of their healthy household contacts. It is not clear what the effect is of the increased ratio of CD3^+^ T lymphocytes on the production of IL-1 family cytokines and IL-10 by PBMCs of NTMPD patients in response to MAC stimulations.

There were some limitations to this study. To avoid interruption by environmental risk factors, we used each patient’s own healthy household contacts as healthy household-matched controls. However, there was a difference in sex between NTMPD patients (15 female) and healthy household contacts (1 female, 14 male). Thus, data may be affected by a difference in sex. The subject number was small, and some significance may have been underestimated. For example, upon stimulation with heat-killed MAC antigens, the production of TNF-α by PBMCs was reduced in NTMPD patients compared with that of their healthy household contacts, but this difference was not statistically significant (*P *= 0.067 upon M. intracellulare stimulation; *P *= 0.054 upon M. avium stimulation) (see Fig. S1 in the supplemental material). All patients and healthy household contacts were Caucasian, and all cases were enrolled in the United States (12 cases from Texas, 2 cases from Louisiana, and 1 case from Arkansas). Thus, the results may not be generalized to other ethnicities or areas. Some patients are currently receiving treatment or had been previously treated with antimycobacterial agents. Thus, data may be affected by treatment with antimycobacterial agents.

In the current study, we found that the defective expression of TLR2 and TWIK2 in NTMPD patients might be involved in reduced cytokine production, which is known to control bacterial growth. Since IL-1 signaling, especially IL-1β, is essential for host defense against mycobacterial infection, it is important to further understand whether defective TLR2 and/or TWIK2 expression or gene polymorphisms cause susceptibility to NTMPD infection or contribute to the development of NTM disease.

## MATERIALS AND METHODS

### Subjects.

Blood samples were obtained from 15 patients with a diagnosis of NTMPD at the University of Texas Health Science Center at Tyler from July 2019 to November 2019. A diagnosis was made on the basis of guidelines recommended by the American Thoracic Society (1). In all cases, pulmonary symptoms (sputum production) were observed, and sputum culture results were positive (5 cases of M. intracellulare, 2 cases of M. avium, 1 case of M. avium and M. abscessus, 3 cases of M. abscessus, 3 cases of Mycobacterium gordonae, and 1 case of Mycobacterium lentiflavum). On chest radiograph, 13 patients had nodular opacities, 1 patient had cavitary opacities, and 1 patient had no radiographic abnormalities. None of the patients had a history of TB and risk factors for HIV infection. At the time that blood samples were obtained, 3 patients had never received treatment, 6 patients were currently receiving treatment for 4 to 17.5 months, and 6 patients had been treated previously for 1 to 24 months at 2 to 48 months ago. All patients were Caucasian females (6 former smokers, 7 who had never smoked, and no current smokers). They had a median age of 65 years (range, 48 to 83 years). To avoid potential perturbation by environmental risk factors, we used the patient’s own healthy household contacts who had not been diagnosed or treated for NTM as a healthy household-matched control. All healthy household contacts were Caucasian (9 former smokers, 6 who had never smoked, and no current smokers). Of 15 healthy household contacts, 1 was female and 14 were male. They had a median age of 68 years (range, 57 to 82 years). Demographics and baseline characteristics of NTMPD patients and their healthy household contacts are shown in Table 1. In some mechanistic studies (Fig. 2B, 4 and 5), blood samples were obtained from healthy volunteers who had not been diagnosed or treated for NTM regardless of age, sex, and race. All studies were performed following the NIH guidelines and regulations, and the protocol was approved by the Institutional Review Board of the University of Texas Health Science Center at Tyler (protocol number 1085). Written informed consent was obtained from all study subjects.

**TABLE 1:** Demographics and baseline characteristics^*a*^

Characteristic	Results for:	
	HHC (*n* = 15)	NTMPD (*n* = 15)
Age (yr)		
Median (range)	68 (57–82)	65 (48–83)
Sex (*n* [%])		
Male	14 (93.3)	0 (0)
Female	1 (6.7)	15 (100)
Race (*n* [%])		
White	15 (100)	15 (100)
Asian	0 (0)	0 (0)
Hispanic	0 (0)	0 (0)
African American	0 (0)	0 (0)
Other	0 (0)	0 (0)
Tobacco use (*n* [%])		
Former smoker	9 (60)	6 (40.0)
Current smoker	0 (0)	0 (0)
Never smoker	6 (40)	9 (60.0)
Sputum culture (*n* [%])		
M. intracellulare	N/A	5 (33.3)
M. avium	N/A	2 (13.3)
M. avium and M. abscessus	N/A	1 (6.7)
M. abscessus	N/A	3 (20.0)
M. gordonae	N/A	3 (20.0)
*M. lentiflavum*	N/A	1 (6.7)
Chest radiograph (*n* [%])		
Nodular	N/A	13 (86.7)
Cavitary	N/A	1 (6.7)
No radiographic lesion	N/A	1 (6.7)
Treatment history (*n* [%])		
Never	N/A	3 (20.0)
Current	N/A	6 (40.0)
Prior	N/A	6 (40.0)

a *n*, number of subjects; HHC, healthy household contact; NTMPD, nontuberculous mycobacterial pulmonary disease; N/A, not applicable; never, patients who had never received treatment; current, patients who are currently receiving treatment; prior, patients who had been previously treated and currently not been treated.

### Bacterial source and stock.

Clinical isolates of M. intracellulare (patient number 16:AF399) and the M. avium Chester (ATCC 700898) were provided by the Department of Microbiology, the University of Texas Health Science Center at Tyler, Texas. The bacteria were cultured and prepared as described in our previous study (40).

### Heat-killed MAC antigens.

M. intracellulare and M. avium were heated at 100°C for 5 min for inactivation as described previously (41). The bacteria were sonicated on ice, and the crude lysates were used as heat-killed MAC antigens. The protein concentration of the MAC antigens was measured using a commercial bicinchoninic acid (BCA) protein assay kit. Heat-killed MAC antigens were resuspended in phosphate-buffered saline (PBS; 5 mg/mL), aliquoted, and stored at −80°C until use.

### PBMC stimulation with heat-killed MAC antigens.

To compare immune responses between NTMPD patients and their own healthy household contacts, PBMCs were isolated from whole blood using density gradient centrifugation with Ficoll-Paque (GE Healthcare). Isolated PBMCs were resuspended at a density of 2 × 10^6^ cells/mL in RPMI 1640 supplemented with 10% heat-inactivated human serum. The cells were then placed in 12-well plates and incubated in the presence or absence of 10 μg/mL heat-killed MAC antigens at 37°C in 5% CO_2_. After a 72-h incubation, the cell-free supernatants were collected, and the cell pellets were treated with TRIzol LS reagent (Invitrogen) and stored at −80°C for subsequent multiplex immunoassay and quantitative real-time PCR.

### Plasma.

Peripheral blood samples were collected into tubes containing sodium heparin and centrifuged at 10,000 × *g* for 10 min at 4°C within 30 min of collection. Plasma samples were collected and stored at −80°C until use.

### Multiplex immunoassay.

A total of 34 cytokines and chemokines were evaluated in the plasma and the cell culture supernatants using commercial multiplex immunoassay kits (34-Plex human ProcartaPlex panel 1A, Invitrogen) according to the manufacturer’s instructions.

### Macrophage stimulation with live M. intracellulare.

Monocyte-derived macrophages of healthy volunteers were prepared (42) and infected with live M. intracellulare at a multiplicity of infection (MOI) of 20 as described previously (43). The cells were further incubated in the presence of various concentrations (0, 100, or 300 ng/mL) of recombinant RANTES (R&D Systems) or plasma from NTMPD patients (10 μL) at 37°C in 5% CO_2_. In some cases, the plasma was pretreated with various concentrations (0, 0.5, or 1 μg/mL) of anti-RANTES antibody (R&D Systems) or 1 μg/mL of isotype control antibody (R&D Systems) at 37°C for 30 min. After 24 h of incubation, cell culture supernatants were collected and stored at −80°C for subsequent cytokine measurements.

### Quantitative real-time PCR.

The mRNA expression of Toll-like receptor 2 (TLR2), P2X7 receptor (P2X7R), *Kcnk2* (encoding TREK1), *Kcnk6* (encoding TWIK2), and *Kcnk12* (encoding THIK2) was measured by real-time PCR using 18S as an internal control with specific primer and probe sets (Applied Biosystems) (see Table S1 in the supplemental material).

### Pharmacological inhibition of TLR2 and two-pore domain K+ channel.

C29 (MedChemExpress) and quinine (Sigma) were used as TLR2-selective inhibitors (44) and two-pore domain K+ channel inhibitor (23, 36), respectively. PBMCs from healthy volunteers were stimulated with 10 μg/mL heat-killed MAC antigens in the presence or absence of various concentrations (10, 50, and 100 μM) of C29 or quinine at 37°C in 5% CO_2_ for 24 h. CD14^+^ monocytes from healthy volunteers were stimulated with live MAC (MOI of 10) in the presence or absence of various concentrations (10, 50, and 100 μM) of C29 or quinine at 37°C in 5% CO_2_ for 18 h. The cell-free supernatants were collected and stored at −80°C until use.

### ELISA and LDH assay.

Commercial ELISA kits were used to determine the amounts of human IL-1β (Mabtech), human IL-18 (R&D Systems), human IL-1α (Biolegend), and human IL-10 (Biolegend) in the culture supernatants of PBMCs and CD14^+^ monocytes. A colorimetric lactate dehydrogenase (LDH) assay kit (Abcam) was used to determine the LDH activity in culture supernatants of PBMCs and CD14^+^ monocytes.

### Flow cytometric analysis.

To compare the ratio of the peripheral blood leukocyte subpopulation between NTMPD patients and their own healthy household contacts, heparinized whole blood (100 μL) was stained with various antibodies (see Table S2 in the supplemental material) for 30 min on ice. Red blood cells were lysed using fluorescence-activated cell sorter (FACS) lysing solution (BD) for 3 min. The cells were washed and resuspended in PBS, and acquisition was performed using an Attune NxT acoustic flow cytometer (Invitrogen). FlowJo software version 10 (BD) was used for flow cytometry data analysis. The gating strategy for leukocyte subpopulations in human whole peripheral blood is shown in Fig. S4 in the supplemental material.

### Statistical analysis.

Data are expressed as the means, and error bars represent SEM for bar graphs (Fig. 1, 2B, 3, 4, 5, and 6; Fig. S1 and S3). Data are also expressed as each individual circle (open circle: healthy household contacts, closed circle: NTMPD patients) and line (median value) for dot plots (Fig. 1, 2A, 3, and 6, Fig. S2). In the case of comparisons between NTMPD patients and their own healthy household contacts (matched control), a paired *t* test or one way analysis of variance (ANOVA) was performed. For statistical analysis involving 3 or more groups, we used one-way ANOVA followed by Fisher’s least significant difference (LSD) test in a *post hoc* manner to evaluate significant differences. Fisher’s LSD test was performed only when the one-way ANOVA test yielded a significant *P* value. A *P* value of <0.05 was considered significant.

## Supplementary Material

Reviewer comments
