# Supplementary material for: Decreased Interleukin-1 Family Cytokine Production in Patients with Nontuberculous Mycobacterial Lung Disease
Source: Microbiol Spectr. 2022 Oct 18;10(6):e03110-22. doi: 10.1128/spectrum.03110-22 (PMC9769609; doi:10.1128/spectrum.03110-22)
Supplement: Supplemental file 1 — Supplemental material. Download spectrum.03110-22-s0001.pdf, PDF file, 0.8 MB [file spectrum.03110-22-s0001.pdf]

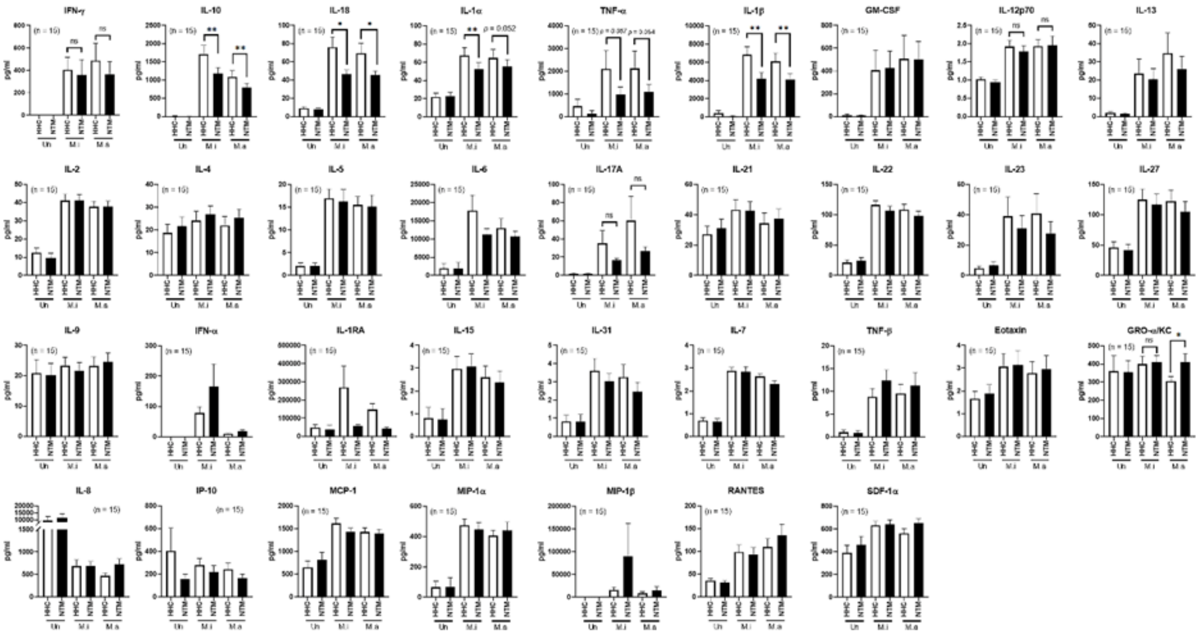

Figure S1 (related to Figure 1): Production of 34 cytokines and chemokines by *M. avium* complex (MAC)-stimulated peripheral blood mononuclear cells (PBMCs). PBMCs were isolated from 15 pairs of NTMPD patients (NTM) and healthy household contacts (HHC) and cultured in the presence or absence of 10 μg/ml heat-killed MAC [*M. intracellulare* (M.i) or *M. avium*(M.a)] for 72 h. The concentrations of 34 cytokines and chemokines were measured by multiplex immunoassay. Data are expressed as the means and error bars represent SEM. *P < 0.05, and **P < 0.01. ns, not significant.

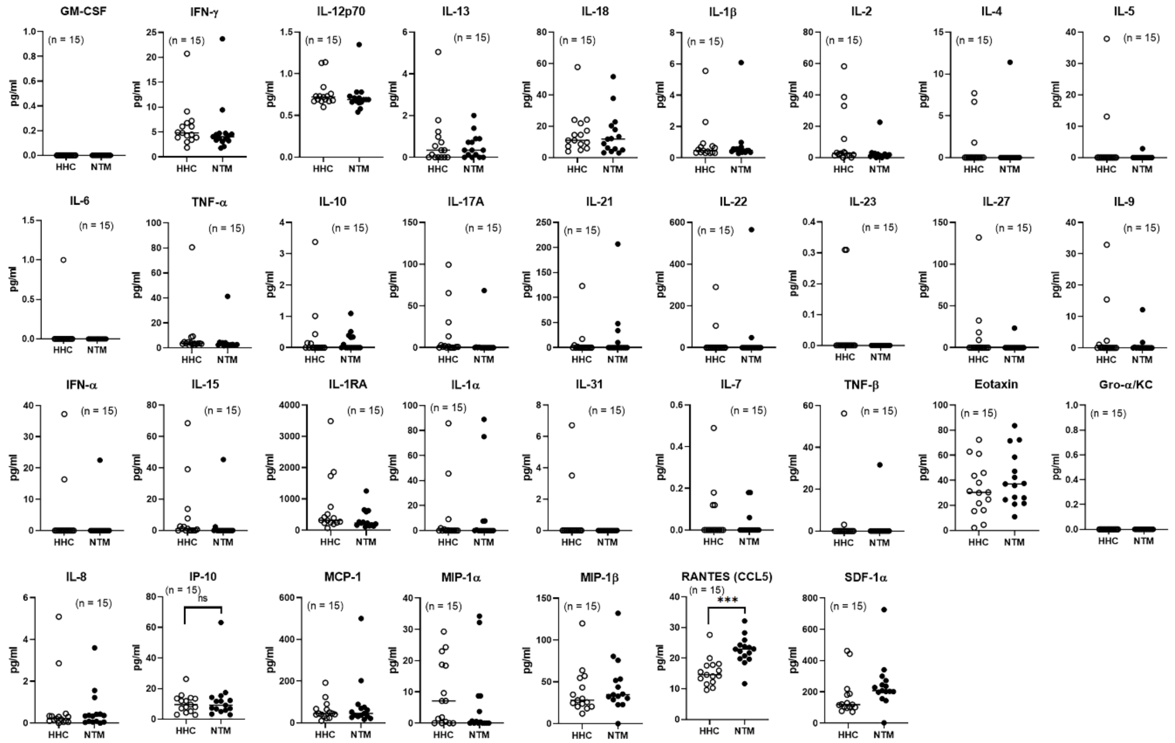

Figure S2 (related to Figure 2): Assessment of 34 cytokines and chemokines in plasma. Plasma samples were collected from 15 pairs of NTMPD patients (NTM) and their own healthy household contacts (HHC). Concentrations of 34 cytokines and chemokines were measured by multiplex immunoassay. Data are expressed as each individual circle (open circle: healthy household contacts, closed circle: NTMPD patients) and line (median value) ***$P < 0.001$. ns, not significant.

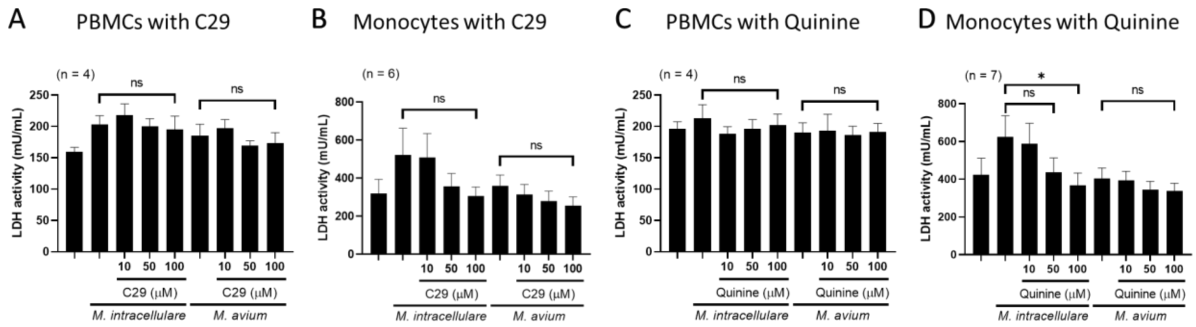

Figure S3 (related to Figure 4 and 5): Lactate dehydrogenase (LDH) activity by PBMCs and CD14+ monocytes. PBMCs were stimulated with 10 µg/ml heat-killed MAC antigens in the presence or absence of various concentrations (10, 50 and 100 µM) of C29 or quinine for 24 h. CD14+ monocytes stimulated with live *M. intracellulare* (MOI of 10) or live *M. avium* (MOI of 10) in the presence or absence of various concentrations (10, 50 and 100 µM) of C29 or quinine for 18 h. A colorimetric LDH assay kit (Abcam) was used to determine LDH activity in PBMCs and CD14+ monocyte culture supernatants. Data are expressed as the means and error bars represent SEM. **P* < 0.05. ns, not significant.

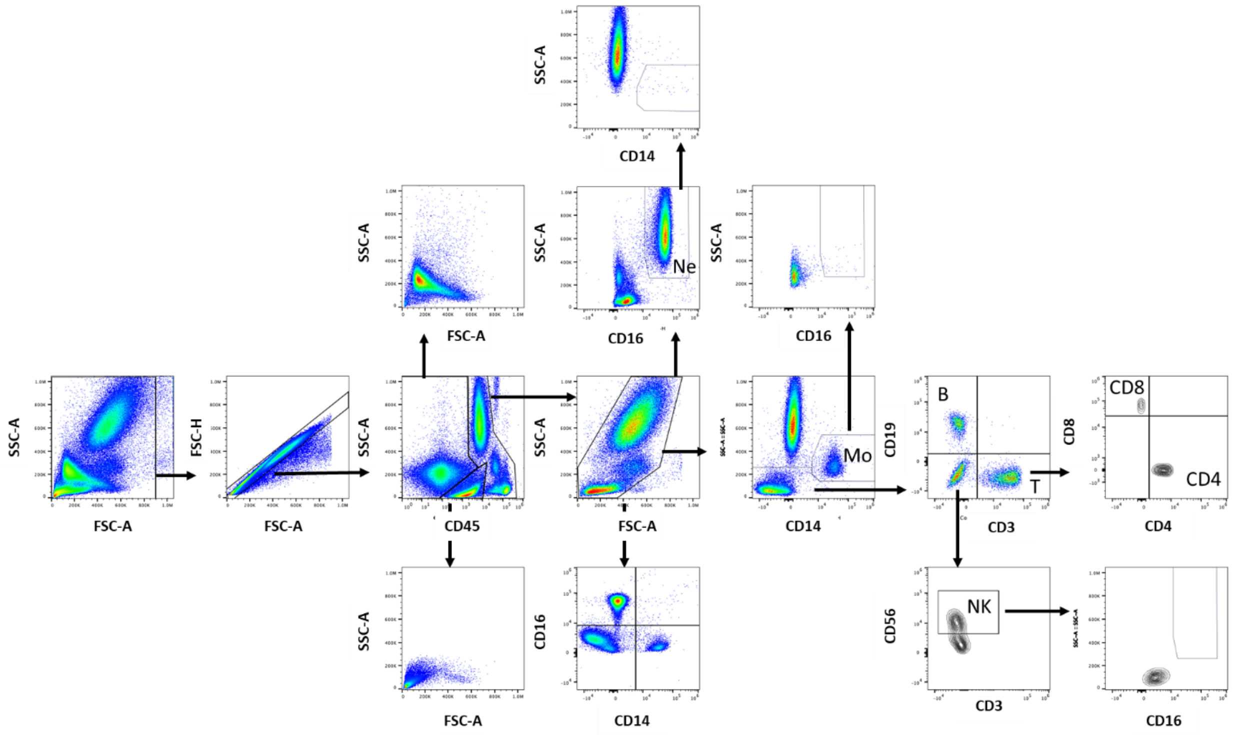

Figure S4 (related to Figure 6): Gating strategy for immune cell phenotyping. Heparinized whole blood was stained with various antibodies (Supplementary Table 1), and immune cells were determined using the illustrated gating strategy. Neutrophils (Ne) were gated as CD45+SSChiCD16+CD14- cells. Monocytes (Mo) were gated as CD45+SSCmidCD16-CD14+ cells. T cells were gated as CD45+SSClowCD3+CD19- cells. B cells were gated as CD45+SSClowCD3-CD19+ cells. CD4 T cells were gated as CD45+SSClowCD3+CD19-CD4+CD8- cells. CD8 T cells were gated as CD45+SSClowCD3+CD19-CD4-CD8+ cells. NK cells were gated as CD45+SSClowCD3- NK1.1+ cells.

Gene Symbol	Gene Aliases	Assay ID (Applied Biosystems)
P2RX7	P2X7	Hs00175721_m1
KCNK12	THIK2	Hs00363726_m1
KCNK2	TREK1	Hs01005159_m1
TLR2	CD282	Hs00152932_m1
18S		Hs99999901_s1
KCNK6	TWIK2	Hs00559239_g1

Table S1 (related to Figure 3): Specific primer and probe sets for real-time PCR analysis.

Antibody	Clone	Dyes	Vendor	Cat#
CD45	H130	Brilliant Violet 421™	Biolegend	304032
CD14	M5E2	Brilliant Violet 510™	Biolegend	301842
CD3	UCHT1	FITC	Biolegend	300406
CD56	HCD56	Brilliant Violet 605™	Biolegend	318334
CD4	OKT4	APC	Biolegend	317416
CD19	HIB19	PerCP/Cyanine5.5	Biolegend	302230
CD16	3G8	Brilliant Violet 711™	Biolegend	302044
CD8	SK1	PE/Cyanine7	Biolegend	344712

Table S2 (related to Figure 6): Antibodies for flow cytometry analysis.
